# Risk of hepatitis C transmission by healthcare workers – a systematic review

**DOI:** 10.3205/dgkh000609

**Published:** 2025-12-18

**Authors:** Roland Diel, Albert Nienhaus

**Affiliations:** 1 Institute of Epidemiology, University Medical Hospital Schleswig-Holstein, Kiel, Germany; 2 Institution for Statutory Accident Insurance and Prevention in the Health and Welfare Services (BGW), Hamburg, Germany

**Keywords:** health care workers, hepatitis C, transmission, infectivity, professional-to-patient, guidelines, exposure prone procedures, opioid diversion

## Abstract

**Background:**

Occupational acquisition of hepatitis C virus (HCV) among healthcare workers (HCWs) has markedly declined in high-income countries, largely due to improved infection control measures and safety-engineered devices. However, the risk of HCV transmission from HCWs to patients remains insufficiently characterized.

**Methods:**

We conducted a systematic review of studies reporting serological evidence of HCV transmission from infected HCWs to patients. Following PRISMA guidelines, we searched MEDLINE, Scopus, and Cochrane databases for publications up to July 2025.

**Results:**

Of 192 studies identified, 24 from eight countries met inclusion criteria. In total, 27 HCWs were implicated as potential sources, and 54,622 patients were tested for HCV RNA. Seventy-six transmissions were confirmed by RNA sequencing; 369 were classified as probable and 15 as possible. Direct provider-to-patient transmission was documented in 18 studies, 12 of which involved exposure-prone procedures (EPP), yielding a mean transmission rate of 0.47% (62/13,224; 95% CI 0.036–0.061%). Viral load data were reported for only eight transmitting HCWs, all with ≥2×10^5^ IU/mL, while no measurements were available for the remaining 19. Given the rarity of direct transmission and the absence of validated HCV RNA thresholds, national guidelines (UK, Germany, the Netherlands, Belgium, France, Switzerland, USA) differ considerably regarding restrictions or monitoring of infected HCWs performing EPP. Indirect HCV transmission associated with diversion of opiates and injection-safety breaches by HCWs addicted to morphine accounted for an even higher risk, with a mean rate of 0.94% (389/41,398; 95% CI 0.85–1.04%).

**Conclusions:**

Evidence on HCW-to-patient HCV transmission remains limited and provides only weak guidance for national prevention policies. Indirect transmission through unsafe practices by opioid diversion has emerged as the predominant pathway, underscoring the need for real-time monitoring, tamper-resistant systems, and strict adherence to infection prevention measures in healthcare settings.

## Zusammenfassung

**Hintergrund:**

Die beruflich bedingte Infektion mit dem Hepatitis-C-Virus (HCV) bei Medizinischem Personal (MP) ist in Ländern mit hohem Einkommen deutlich zurückgegangen, vor allem aufgrund verbesserter Infektionskontrollmaßnahmen und sicherheitsoptimierter Instrumente. Das Risiko einer HCV-Übertragung von MP auf Patienten ist jedoch nach wie vor unzureichend charakterisiert.

**Methoden:**

Wir führten ein systematisches Review von Studien durch, die serologische Nachweise einer HCV-Übertragung von infiziertem MP auf Patienten berichteten. Unter Beachtung der PRISMA-Richtlinien durchsuchten wir bis Juli 2025 die Datenbanken MEDLINE, Scopus und Cochrane nach einschlägigen Publikationen.

**Ergebnisse:**

Von 192 identifizierten Studien erfüllten 24 aus acht Ländern die Einschlusskriterien. Insgesamt wurden 27 Beschäftigte im Gesundheitswesen als mögliche Infektionsquellen (Indexpersonen) identifiziert, und 54.622 Patienten wurden auf HCV-RNA getestet. 76 Übertragungen wurden mittels RNA-Sequenzierung bestätigt; 369 wurden als wahrscheinlich und 15 als möglich eingestuft. Eine direkte Übertragung von MP auf Patienten wurde in 18 Studien dokumentiert, von denen 12 expositions-risikobehaftete Eingriffe (exposure-prone procedures, EPP) beinhalteten. Daraus ergab sich eine mittlere Übertragungsrate von 0,47% (62/13.224; 95%-KI 0,036–0,061%). Viruslastdaten lagen lediglich für acht übertragende Indexpersonen vor, allesamt mit ≥2×10^5^ IU/ml, während für die übrigen 19 keine Messungen verfügbar waren. Angesichts der Seltenheit direkter Übertragungen und des Fehlens validierter HCV-RNA-Schwellenwerte unterscheiden sich die nationalen Richtlinien (UK, Deutschland, Niederlande, Belgien, Frankreich, Schweiz, USA) erheblich in Bezug auf Einschränkungen oder Monitoring von infiziertem MP bei EPP. Indirekte Übertragungen im Zusammenhang mit Medikamentendiebstahl und Verstößen gegen Injektionssicherheit von morphinabhängigem Personal waren bei einer mittleren Rate von 0,94% (389/41.398; 95%-KI 0,85–1,04%) mit einem noch höherem Risiko verbunden.

**Schlussfolgerungen:**

Die Evidenz zur HCV-Übertragung von MP auf Patienten bleibt begrenzt und liefert nur eine schwache Grundlage für nationale Präventionsstrategien. Indirekte Übertragungen durch unsichere Praktiken bei Opiatabzweigung haben sich als Hauptübertragungsweg herausgestellt. Dies unterstreicht die Notwendigkeit eines Echtzeit-Monitorings, manipulationssicherer Systeme sowie der konsequenten Einhaltung von Infektionsschutzmaßnahmen im Gesundheitswesen.

## Introduction

Hepatitis C virus (HCV) is a blood-borne pathogen transmitted mainly through parenteral exposure, including transfusions, unsafe injections, and occupational needlestick injuries. It remains a major cause of chronic liver disease worldwide, leading to cirrhosis, hepatocellular carcinoma, and liver-related mortality. Despite the advent of highly effective direct-acting antivirals (DAAs), which achieve cure rates exceeding 95%, HCV continues to pose a global health threat. The World Health Organization (WHO) estimates that approximately 50 million individuals are currently living with chronic HCV infection, with about 1 million new infections and 242,000 deaths annually. Importantly, no vaccine is available against HCV, rendering prevention efforts dependent on blood safety, harm reduction, and rigorous infection prevention and control (IPC) measures in healthcare settings [1].

Occupational acquisition of HCV among healthcare workers (HCWs) following patient exposure is well documented [2], but in high-income settings incidence has fallen markedly over the past two decades. German accident-insurance data demonstrate a sustained decline in recognised occupational hepatitis C cases: the Institution for Statutory Accident Insurance and Prevention in the Health and Welfare Services (BGW) reported a reduction from 692 cases of HBV and HCV combined during 1996–2000 to only 99 cases in 2016–2020, of which 59 were HCV. This represents an 85.7% reduction [3]. In the subsequent period 2021–2023, only two new occupationally acquired HCV cases were recognized nationwide [4].

Comparable trends are seen internationally. In the United Kingdom, national surveillance (“Eye of the Needle”) has documented only a handful of HCV seroconversions in recent years, with the last confirmed case following an exposure in 2015 [5]. In the United States, occupational risk has also declined significantly. A recent longitudinal study of 885 HCWs with percutaneous exposures to HCV-positive blood reported a seroconversion rate of just 0.2%, compared with earlier CDC estimates of approximately 1.8% in similar scenarios [6]. These data reflect both improved improved infection prevention and control (IPC) measures and widespread adoption of safety-engineered devices. A 2025 multi-hospital study further confirmed that although sharps injuries remain frequent, no HCV, HBV, or HIV seroconversions were observed during follow-up [7]. Together, these findings indicate that patient-to-provider transmission of HCV has become exceedingly rare in modern healthcare systems.

Against this backdrop of declining occupational acquisition, the reverse route of transmission—from HCV-infected providers to their patients—deserves critical attention. In contrast to HBV, such cases have been reported only rarely. No HCW-to-patient transmissions were published prior to 1995, when a bulletin report from England first suggested a link between acute HCV in a cardiac surgery patient and exposure to an HCV-infected healthcare worker [8]. While this initial report was based only on epidemiological association, molecular evidence followed soon after: in 1996, a Spanish outbreak investigation described probable transmission from an HCV-infected surgeon to five patients [9], and in 1997 Duckworth et al. provided the first sequencing-confirmed case of HCW-to-patient transmission in a retrospective cohort of 278 cardiac surgery patients [10]. Since then, a gradually growing number of case reports, bulletin notices, and retrospective investigations have documented such events and have occasionally been summarised in narrative reviews (e.g. [11], [12], [13]).

Surprisingly, despite the clinical and ethical importance of this issue, no systematic review has so far comprehensively addressed HCV transmission from infected HCWs to patients, nor the evidence base for restricting clinical practice in such cases. The present review therefore seeks to critically appraise the published literature on HCW-to-patient HCV transmission, with particular focus on modes of transmission and the impact of reported cases on national and international guideline development.

## Methods

### Definition of HCWs

HCWs were defined as all medical, dental, nursing, obstetric or assisting personnel working in different areas, e.g. hospitals, outpatient clinics, doctors' practices, dialysis facilities, nursing homes and out-patient care facilities. The decisive factor was the existence of a plausible transmission pathway within these activities.

### Literature search and study selection

A comprehensive literature search up to July 1, 2025, was conducted in PubMed, the Cochrane Library, and Google Scholar to identify reports of HCV transmission from healthcare workers (HCWs) to patients. Search strategies were adapted to the indexing systems and functionalities of each database to maximise both sensitivity and precision. Full search strings for each database, together with the methodological rationale for their design, are presented in Attachment 1. Only studies or reports written in English language published that provided original serological data on suggested nosocomial HBV transmission to patients were considered, without restriction to publication date. 

Review articles, guidelines, conference abstracts, newspaper articles, press releases, commentaries, editorials, studies without a defined HCV source and articles with a central theme diverging from or not related to reported professional-to-patient transmission of HCV were excluded. No restrictions were applied regarding study design, patient subpopulation, or mode of data collection (prospective or retrospective). If studies reported preliminary findings, the most complete and up-to-date and complete data version of the data was used. 

Reference lists of the included articles as well as of the review articles were manually screened to identify additional eligible publications. All records were managed using EndNote which automatically removed duplicates. The preferred reporting items for systematic reviews and meta-analysis (PRISMA) standards 2020 guidelines were followed [14], [15]. 

### Data extraction

Relevant studies were independently selected by two reviewing authors (RD and AN), who screened each article title and abstract initially, and then went on to review an article’s full text as required. Any discrepancies were resolved by consensus. The following variables were recorded where available:

1. country and year of publication;
2. study period;
3. study design;
4. occupation or workplace of the suspected source HCW;
5. number of persons tested (including staff and any secondary HCV cases identified);
6. HCV genotype (if available);
7. viral load of the source case (IU/mL, or genome equivalents per mL where applicable);
8. number of transmissions (classified as confirmed, probable, or possible);
9. suspected transmission route. HCV viremia levels were reported either as RNA copies/mL or IU/mL, with five HCV RNA copies corresponding to approximately one IU.

Studies were categorised as direct transmission (HCW-to-patient contact) or indirect transmission (exposure via contaminated products or systems without direct contact). Furthermore, studies were evaluated for the performance of exposure-prone procedures (EPPs), defined as procedures in which the HCW’s hands or fingers are within poorly visualised or confined body sites, with a significant risk of injury and subsequent contact with the patient’s open tissues. We deliberately refrained from conducting a formal meta-analysis and summarised the findings descriptively, as the primary aim of this review was to provide a systematic overview and appraisal of the available evidence.

### Definition of transmission probability

Currently, there are no universally accepted definitions regarding the classification of HCW-to-patient HCV transmission. For this review, we applied a uniform classification to ensure consistency across studies. In all cases, epidemiological links (documented exposure during healthcare procedures, temporal association, and exclusion of alternative infection sources) were required. Based on this prerequisite, molecular confirmation defined the level of certainty:

- a) Confirmed transmission: Epidemiological evidence plus complete or near-complete genetic identity between HCW and patient viral sequences, demonstrated by full-genome or high-resolution sub-genomic sequencing (e.g., E1/E2, NS5B). Minor intrahost quasi-species variation was accepted.
- b) Probable transmission: Epidemiological evidence plus high but not absolute genetic relatedness between HCW and patient, typically clustering within the same phylogenetic branch with strong bootstrap/posterior support, or >90–95% sequence homology in key genomic regions.
- c) Possible transmission: Epidemiological evidence with either no molecular data or results showing only moderate relatedness.

Studies reporting identical genotypes between index cases and infected patients but lacking detailed sequencing results were classified as probable.

### Assessment of study quality

All of the studies included under these criteria were retrospective observational studies or case reports designed either to identify either the source of HCV infection or to detect secondary cases. Since test results were highly dependent on patient record availability, the chosen observation period, laboratory capacity, and patient willingness to undergo serological testing, the data were prone to both selection and information bias. A formal assessment of study quality, e.g. by using the Joanna Briggs Institute (JBI) critical appraisal of prevalence studies scale [16] was therefore not deemed appropriate.

## Results

### Study availability

**Figure 1:**
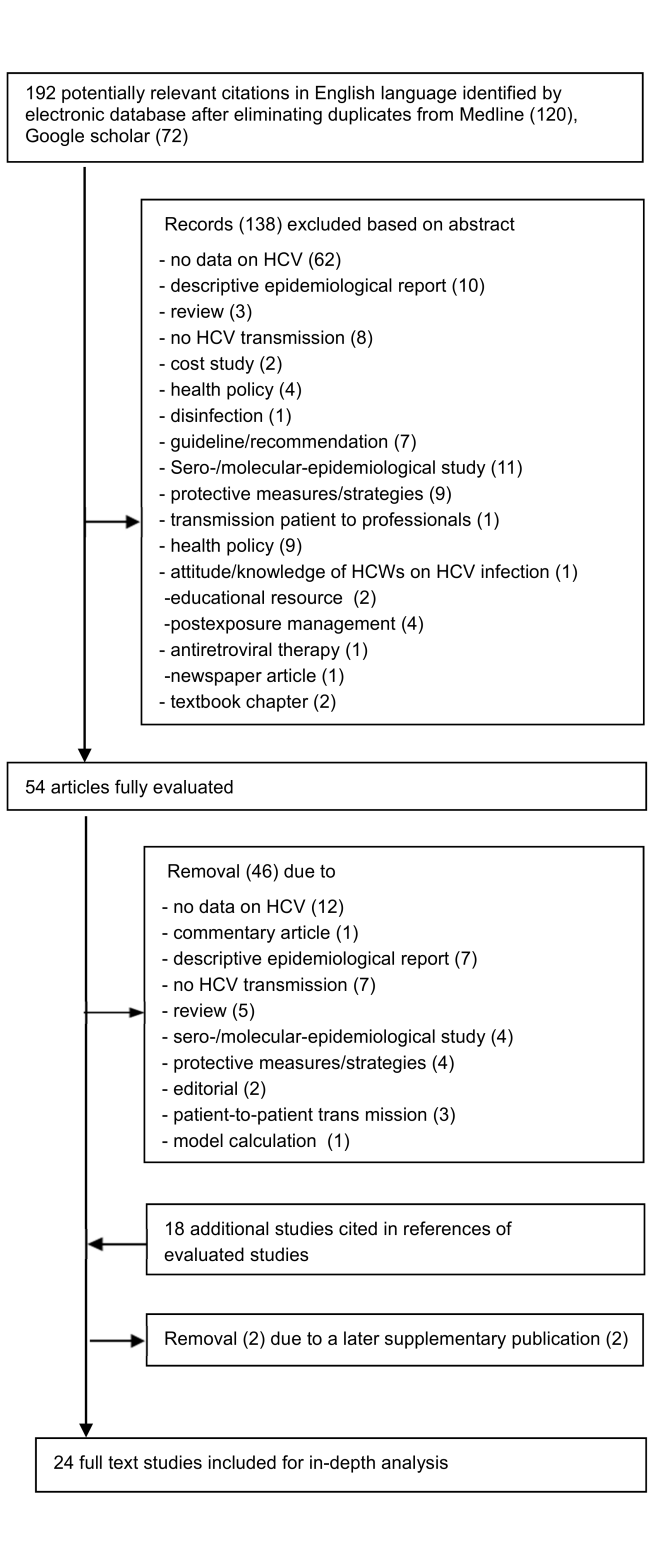
PRISMA flow diagram of study selection

Figure 1 (Fig. 1) shows the flow diagram of the literature search. In total, 192 abstracts in English were identified (120 in PubMed and 72 in Google Scholar), with reviews being excluded by default in the search strategy. The search of the Cochrane Library using the predefined strategy retrieved no records. After exclusion of 138 records based on their abstracts, 54 full-text articles were reviewed. Of these, 8 studies met the eligibility criteria. An additional 18 studies, not captured by the search strategy, were identified through reference lists of full-text articles. Two bulletin reports from the UK Communicable Disease Report (CDR) Weekly [8], [17] were replaced by the studies of Duckworth [10] and Perry [11], which provided updated data on the number of patients tested and sequencing methods. In total, 24 peer-reviewed studies [9], [10], [11], [18], [19], [20], [21], [22], [23], [24], [25], [26], [27], [28], [29], [30], [31], [32], [33], [34], [35], [36], [37], [38] were included in the analysis.

### Study characteristics

The characteristics of the included studies are summarised in Table 1 (Tab. 1) and Table 2 (Tab. 2). All 24 studies originated from high-income countries and were published between 1995 and 2018. Most were conducted in the USA (7/24, 29.2%), the UK (6/24, 25.0%), and Germany (4/24, 16.7%). Spain and France each contributed two studies (8.3%), while Australia, Israel, and Norway each contributed one (4.2%).

### Study design

Fifteen of the 24 studies (62.5%) were retrospective observational investigations, six (25%) were retrospective cohort studies comparing exposed with unexposed individuals, and three were case series or case reports. A total of 54,622 patients underwent serological testing for HCV RNA. Altogether, 451 cases of HCW-to-patient transmission were reported: 76 were confirmed by sequencing, 360 were considered probable, and 15 were classified as possible after exclusion of alternative risk factors. Overall, a mean transmission rate of 0.47% was observed (62/13,224; 95% CI 0.036–0.061%). Study sample sizes ranged from 1 to 28,618 participants.

### Index cases

In 12 studies, transmissions were attributed to surgeons of various specialties performing EPPs. In four studies, the source was an anaesthesiologist; in one study, a home-care nurse; and in another, a midwife. Only two studies documented specific hand injuries in the transmitting HCW, whereas in 16 of 18 studies involving direct transmission, no obvious mechanism was identified. It is noteworthy that the exposure period of the reported possible direct transmissions related to EPP ended in September 2008 [31].

In six studies, HCV transmission occurred indirectly, not through accidental intraoperative or nursing exposures, but via deliberate drug diversion of opioids by HCWs addicted to morphine (Table 2 (Tab. 2)). Patients were exposed when residual contents of contaminated syringes were reused, thereby introducing infected blood into injection equipment. In these investigations, 389 patients were infected out of 41,398 tested, corresponding to a mean transmission rate of 0.94% (95% CI 0.85–1.04%).

### Serological markers and viral load

HCV RNA load was determined for only eight infected HCWs, and in all cases the values exceeded 2×10^5^ IU/mL (Table 1 (Tab. 1)). No viral load data were available for the remaining 19 HCV-positive index cases.

### Translation of the results of HCW transmission studies into national guidelines

In light of our findings, it should be noted that EPPs play only a subordinate role in the direct transmission of HCV to patients. While international guidelines on iatrogenic prevention of HBV are increasingly converging [39], the consistently low risk of HCV transmission to patients has led to markedly different conclusions across countries. There is no universal policy imposing uniform restrictions on HCV-infected healthcare workers performing EPPs. Instead, European and several national guidelines emphasise professional responsibility, medical supervision, and risk minimisation rather than blanket exclusions.

The European guidelines [40] do not recommend prohibiting EPPs for HCV-infected HCWs. However, as a minimum requirement, all HCWs performing EPPs should be aware of their HCV status and be referred to a hepatologist for treatment, with the aim of reducing transmission risk to patients. In the Netherlands [41], the Commission report of 12 December 2015 made no explicit recommendations. Infected HCWs are not prohibited from working but receive advice from a qualified expert on safe working practices, optimal reporting of needlestick injuries, and available treatment options. Belgium [42] applies a case-by-case approach, considering treatment status, preventive measures, and expert consultation. Physicians must adhere to strict preventive measures and may voluntarily adapt or restrict their practice. Where disagreement arises regarding necessary precautions, an expert panel or the provincial medical commission determines the degree of infectiousness and professional fitness.

In France [43], HCV infection itself is not regarded as grounds for professional unfitness or restriction, provided adequate precautions are observed. Infected HCWs must know their serological status; those who are HCV RNA positive are advised to consult a specialist and initiate antiviral therapy. If treatment fails, referral to a special commission is required to consider practice modification or professional reorientation. Regular serological testing is recommended for HCWs in high-risk specialties, such as surgery.

According to Swiss guidelines [44], HCWs performing EPPs should be referred to a “consulting expert group” to evaluate the case and provide individual recommendations. Performing EPPs is not contraindicated, although retraining for work without EPPs should be discussed.

The most restrictive position is taken in the current UK guidelines [45]. According to the Advisory Panel for HCWs Infected with Bloodborne Viruses (UKAP), HCWs with HCV antibodies who are HCV RNA negative may perform EPPs, while those who are HCV RNA positive are restricted. HCWs who spontaneously clear the infection and remain HCV RNA negative for at least three months, as well as those who achieve a sustained virologic response (SVR) for at least three months after antiviral therapy, may resume EPPs. As a safeguard, both groups must undergo confirmatory testing three months after resuming EPPs.

Also in Italy [46], HCWs with confirmed HCV infection may only perform EPPs if serum HCV RNA remains undetectable in three-monthly testing. HCWs who abstain from invasive procedures are not considered to pose a transmission risk, regardless of infection status.

In the United States [49], guidelines have shifted from relatively flexible to significantly stricter. The 2010 SHEA guidelines [12] proposed a maximum HCV viral load threshold of 10^4^ genome equivalents (≈2,000 IU/mL) for HCWs performing EPPs. This threshold was not evidence-based but extrapolated from HBV guidance, where viral load clearly correlates with transmission risk. For HCV, the authors explicitly acknowledged that no empirical data supported such a threshold, rendering it essentially arbitrary. The updated 2022 SHEA guidance [47] substantially revised this approach. Henderson et al. now recommend that HCV-infected HCWs demonstrate undetectable HCV RNA—i.e., a sustained virologic response (SVR) after direct-acting antiviral (DAA) therapy—as a prerequisite for performing EPPs. In rare cases where SVR is not achieved (treatment failure), the pragmatic threshold of 2,000 IU/mL from the 2010 guideline is still mentioned, though the authors stress that no viral load cut-off reliably predicts transmission risk.

Germany has taken an intermediate stance. The 2020 DVV recommendations [48] state that HCV-infected HCWs should not be excluded from their profession in general. Adopting the 2010 US SHEA guidance, HCV RNA levels above 10^5^ copies/mL (≈20,000 IU/mL) are considered incompatible with EPPs. Conversely, HCWs with viral loads ≤10³ copies/mL (≈200 IU/mL) are not restricted from EPPs or other duties, though they should undergo viral load testing every three months. Sustained RNA negativity three months after treatment completion is considered a cure. HCWs with intermediate viral loads (200–20,000 IU/mL) should only perform procedures with a lower risk of transmission, such as endoscopic or laparoscopic interventions.

## Discussion

To our knowledge, this analysis represents the first systematic review of published reports on HCV transmission from HCW to patients. Across 24 studies with serological test results spanning 25 years (1993–2018), only 76 confirmed and 320 probable transmissions were identified, along with 15 possible cases, out of a tested population of 54,622 patients. The resulting unweighted mean rate of 0.047% highlights the rarity of such events. (Tab. 2)

As shown in our compilation (Table 1 (Tab. 1)), however, the number of HCV transmissions associated with EPPs was not evenly distributed but occurred predominantly in the 1990s through the mid-2000s. The last reported exposure period of a published EPP-related transmission ended in September 2008 with the study by Bourigault et al. [31]. This abrupt decline may plausibly be related to improved IPC measures, as seen for occupationally acquired HCV infections overall.

The challenge of deriving national guidelines from these data is further compounded by the fact that, in most published studies, viral load determinations for HCV-infected HCWs were either not performed or not reported. In the eight cases where viral load was quantified, values exceeded 2×10^5^ IU/mL, suggesting that high-level viremia may contribute to transmission. However, the limited dataset precludes the establishment of a reliable viral load threshold—unlike HBV, where quantitative cut-offs are well defined. Consequently, guideline recommendations remain heterogeneous across countries.

While the UK, USA, and Italy require undetectable HCV RNA as a prerequisite for performing exposure-prone procedures (EPPs), most Western European countries generally do not prohibit HCV-infected HCWs from performing such procedures. Instead, they emphasise the provider’s responsibility to initiate antiviral therapy or delegate decisions on professional fitness to institutional committees. Germany historically defined a threshold of 250 IU/mL, derived from the now-abandoned US recommendations of 2010, which themselves—despite limited evidence—had extrapolated this value from HBV guidance.

This heterogeneity reflects both the scientific uncertainty and the policy dilemmas in balancing patient safety with the non-discriminatory treatment of HCWs. Today, however, highly effective short-course DAA therapies are available, achieving overall SVR12 rates of up to 98%, and can therefore rapidly resolve the issue of any potential work restrictions [49].

More concerning than the risk of direct HCV transmission during EPPs—documented in only 14 of 18 studies involving direct provider-to-patient contact—is indirect transmission. In six outbreak investigations since 1998, infections were traced to HCWs who diverted injectable narcotics for personal use, thereby contaminating patient syringes through tampering, swapping, or refilling. This scenario highlights an alternative but clinically significant transmission pathway, independent of surgical or invasive procedures. Exposing patients to blood from an HCW in this context represents an equally serious risk, with the potential to affect large numbers of patients. Indeed, at least 41,398 exposed patients were identified in these outbreaks, among whom 389 confirmed or probably linked infections occurred—corresponding to a mean transmission rate of 0.94%, approximately double that observed after direct exposure (Table 2 (Tab. 2)).

One critical methodological caveat relates to the denominator used in estimating transmission rates. For example, the frequently cited study by Schäfer et al. [36] included patients who were considered exposed, but not all were actually tested. This suggests that the true denominator of tested patients may have been smaller, implying that the actual transmission rate could be higher than reported.

## Conclusions

Our review suggests that the risk of direct provider-to-patient HCV transmission is very low. However, indirect transmission through unsafe injection practices emerged as an equally important—if not more significant—pathway than direct transmission during surgery. This finding underscores the essential role of structural and behavioural safeguards within healthcare institutions and highlights the need for systematic monitoring, tamper-evident safety systems, and robust institutional responses to addiction-related risks among healthcare personnel.

## Notes

### Authors’ ORCIDs

- Diel R: https://orcid.org/0000-0001-8304-7709
- Nienhaus A: https://orcid.org/0000-0003-1881-7302

### Competing interests

The authors declare that they have no competing interests.

## Supplementary Material

Search strategies

## Figures and Tables

**Table 1:**
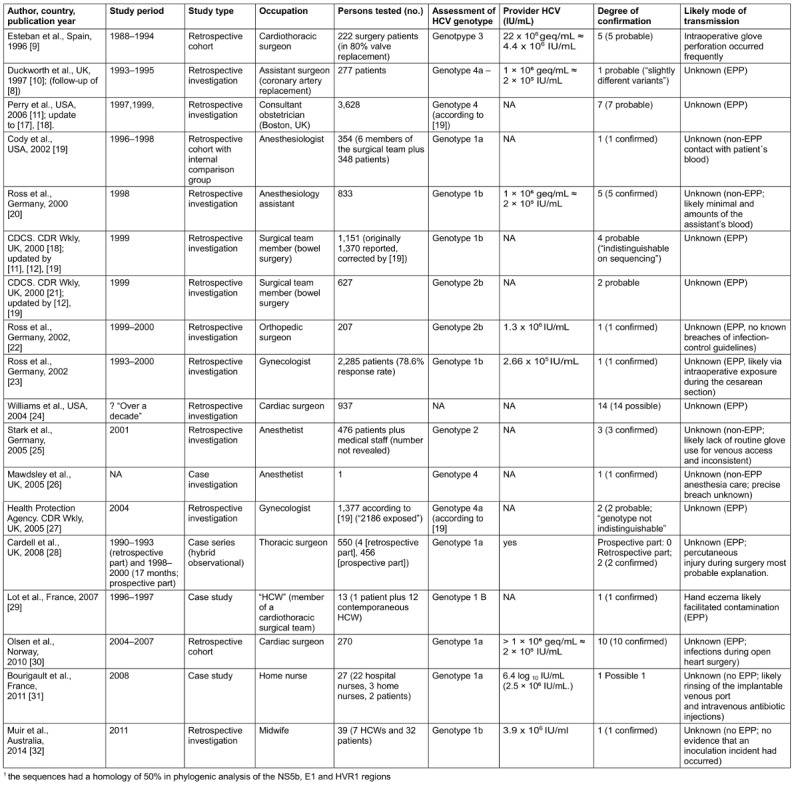
Results of suggested direct HCV transmission by HCW

**Table 2:**
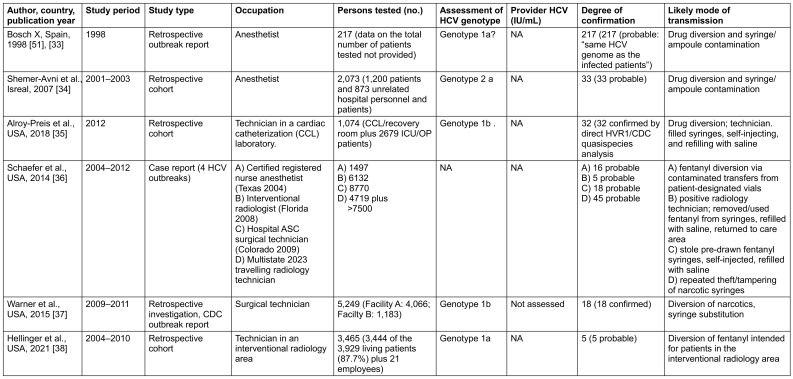
Results of suggested indirect HCV transmission by HCW
