# Supplementary material for: Risk of hepatitis C transmission by healthcare workers – a systematic review
Source: GMS Hyg Infect Control. 2025 Dec 18;20:Doc80. doi: 10.3205/dgkh000609 (PMC12720405; doi:10.3205/dgkh000609)
Supplement: Search strategies [file HIC-20-80-s-001.pdf]

Attachment 1

Search strategies

Although both databases use MeSH indexing, the Cochrane Library requires different field tags, proximity operators, and filter syntax than PubMed, necessitating structural adaptations of the search string;

For the PubMed search, a hybrid syntax was constructed to combine indexed literature (MeSH-based) with non-indexed literature (free-text). This approach ensures inclusion of articles correctly indexed under established subject headings (improving precision) and captures newer articles not yet indexed, as well as those using alternative terminology (improving sensitivity). The search was tailored to the desired transmission direction — healthcare worker (HCW) to patient — by using direction-specific free-text terms such as “professional-to-patient”, “provider-to-patient”, and “iatrogenic transmission”. Terms such as “occupational exposure” or “needle-stick”, which typically refer to patient-to-HCW transmission, were excluded to minimise irrelevant retrieval. To prioritise retrieval of primary reports most relevant to HCW-to-patient transmission scenarios, the keywords “case reports” and “outbreak” were included:
("Hepatitis C"[MeSH Terms] OR "hepatitis C"[All Fields]) AND ("Health Personnel"[MeSH Terms] OR "healthcare worker"[All Fields] OR "health care worker"[All Fields] OR "medical staff"[All Fields]) AND ("Disease Transmission, Infectious"[MeSH Terms] OR (infectious[All Fields] AND transmission[All Fields])) AND ("professional-to-patient"[All Fields] OR "provider-to-patient"[All Fields] OR "healthcare worker-to-patient"[All Fields] OR "surgeon-to-patient"[All Fields] OR "iatrogenic transmission"[All Fields]) NOT ("needle-stick"[All Fields] OR "occupational exposure"[All Fields] OR "patient-to-healthcare worker"[All Fields]) NOT (review[pt] OR systematic[sb]) AND ("Case Reports"[pt] OR outbreak[All Fields] OR "Evaluation Study"[pt]).

In contrast to PubMed, Google Scholar does not use controlled vocabulary indexing; instead, it searches the full text, abstracts, and metadata. In this context, the term “occupational exposure” is applied more variably, including in descriptions of occupationally acquired infection in patients following exposure to an infected HCW. Retaining the term in the Google Scholar strategy therefore increased the likelihood of identifying relevant reports and enhanced search sensitivity: “hepatitis C" AND ("surgeon" OR "dentist") AND ("iatrogenic" OR "professional-to-patient" OR "provider-to-patient" OR "occupational exposure") AND ("outbreak" OR "case report") -"needle-stick" -"sharps injury" -"patient-to-healthcare worker" -"review" -"systematic review" -"literature review" -"mouse" -"mice" -"rat" -"dog" -"animal study".

While both PubMed and the Cochrane Library use MeSH indexing, the Cochrane Library applies a different search syntax, including specific field tags (e.g., :ti,ab,kw for title, abstract, and keywords) and proximity operators (e.g., NEAR/n). Cochrane also allows direct language (english:la) and publication type filters (review:pt) within the search string, which are implemented differently in PubMed. These technical differences required adaptation of the search structure and operators while retaining the same conceptual search blocks: (“Hepatitis C” OR [mh “Hepacivirus”]) AND (“healthcare worker*” OR “health care worker*” OR [mh “Health Personnel”] OR “medical staff” OR “surgeon*” OR “dentist*”) AND ([mh “Disease Transmission, Infectious”] OR [mh “Cross Infection”] OR [mh “Iatrogenic Disease”] OR iatrogenic OR “provider-to-patient” OR “professional-to-patient” OR “healthcare worker-to-patient” OR “surgeon-to-patient” OR nosocomial OR (transmi* NEAR/3 (infect* OR disease OR virus OR HCV))) AND (“outbreak*” OR “case report*” OR “incident investigation” OR “contact tracing”) AND (english:la) NOT (review:pt OR “systematic review” OR “literature review”).

Attachment 1 to: *Diel R, Nienhaus A. Risk of hepatitis C transmission by healthcare workers – a systematic review. GMS Hyg Infect Control. 2025;20:Doc80. DOI: 10.3205/dgkh000609*
